# Impact prediction of translocation of the mitochondrial outer membrane 70 as biomarker in Alzheimer's disease

**DOI:** 10.3389/fnagi.2022.1013943

**Published:** 2022-11-02

**Authors:** Xi Cao, Yanting Chen, Xiaoyu Sang, Shunliang Xu, Zhaohong Xie, Zhengyu Zhu, Ping Wang, Jianzhong Bi, Linlin Xu

**Affiliations:** Department of Neurology, The Second Hospital of Shandong University, Jinan, China

**Keywords:** Alzheimer's disease, TOM70, gene expression, progression, biomarker

## Abstract

Mitochondrial dysfunction plays a key role in the pathogenesis of Alzheimer's disease (AD). The translocase of the outer membrane (TOM) complex controls the input of mitochondrial precursor proteins to maintain mitochondrial function under pathophysiological conditions. However, its role in AD development remains unclear. TOM70 is an important translocase present in the TOM complex. In the current study, we found that TOM70 levels were reduced in the peripheral blood and hippocampus of the APP/PS1 mice. In addition, we examined the whole-blood mRNA levels of TOM70 in patients with AD, dementia with Lewy bodies (DLB), and post-stroke dementia (PSD). Our study revealed that the mRNA level of TOM70 was decreased in the blood samples of patients with AD, which was also correlated with the progression of clinical stages. Therefore, we proposed that the expression of TOM70 could be a promising biomarker for AD diagnosis and monitoring of disease progression.

## Introduction

Alzheimer's disease (AD) is a neurodegenerative disease characterized by progressive cognitive and behavioral impairments. According to recent epidemiological statistics worldwide, AD is the most common cause of dementia, and it is estimated that more than 50 million people worldwide are currently living with dementia. This number is expected to exceed 150 million by 2050 (Lynch, 2020). However, AD lacks effective treatments. Current drugs can only relieve symptoms but do not prevent neuronal loss, brain atrophy, and consequent progressive cognitive deterioration (Zhao, 2020). In addition, methods for diagnosing AD and determining its progression are still limited. Accurate biomarker diagnosis requires expensive infrastructure such as positron emission tomography (PET) scanners or cerebrospinal fluid facilities. In contrast, blood-based biomarkers are non-invasive, inexpensive, and widely available. Therefore, it is necessary to develop novel blood biomarkers for AD diagnosis.

According to the current knowledge, the mechanism of AD remains unclear. However, recent studies have indicated that mitochondrial dysfunction plays an important role in the pathogenesis and progression of AD. Mitochondria are the key organelles that control hemostasis during cell respiration. The logical link between AD and mitochondrial dysfunction has been established for decades. Studies have shown that mitochondria are dysfunctional in AD, including decreased mitochondrial biosynthesis (Song et al., 2018), abnormal mitochondrial dynamics (Xu et al., 2017; Flannery and Trushina, 2019), and mitophagy dysfunction (Cai and Jeong, 2020; Song et al., 2021). Although mitochondria have their own DNA, most of the proteins that mitochondria need are encoded by nuclear genes and are synthesized in the form of precursor proteins with mitochondrial targeting signals in the cytoplasmic ribosome, which are recognized and transported to the mitochondria by TOM complex, to perform their corresponding physiological functions (Chacinska et al., 2009; Mårtensson et al., 2019).

TOM70, one of the main receptor proteins of the TOM complex, is an important component of the TOM complex (Waller et al., 2009; Heinz and Lithgow, 2013) that functions to recognize precursor proteins (Brix et al., 1997; Käser et al., 2016; Backes et al., 2018). A recent study has shown that TOM70 is involved in mitochondria not only as a recognition receptor for mitochondrial precursor proteins but also as a key regulatory protein in the mitochondrial compromised protein import response (mitoCPR) (Weidberg and Amon, 2018). In this study, we found that TOM70 levels were decreased in the peripheral blood of AD patients, but did not change in DLB and PSD patients. Based on the above studies, we speculated that TOM70 may undergo dynamic changes in AD or may be used as a peripheral blood biomarker to diagnose AD and judge its progression.

## Materials and methods

### Participants

Patients with AD, DLB, PSD, and age- and sex-matched cognitively normal controls were enrolled from the Second Hospital of Shandong University. Cognitive function was assessed using neuropsychological tests including the Mini-Mental State Examination (MMSE), Montreal Cognitive Assessment (MoCA), and Clinical Dementia Rating (CDR). AD was diagnosed in accordance with the recommendations of the National Institute on Aging-Alzheimer's Association workgroups on diagnostic guidelines (Jack et al., 2011). DLB was diagnosed according to the 2017 DLB diagnostic guidelines (McKeith et al., 2017). PSD was diagnosed in accordance with the 2017 post-strike dementia comprehensive review (Mijajlovi et al., 2017). MRI T1-weighted images were used to investigate regional brain atrophy by using the medial temporal lobe atrophy scale (MTA) (Scheltens et al., 1992). All the participants signed an informed consent form. All procedures were performed in accordance with the Declaration of Helsinki.

### Animal model

Male APP/PS1 transgenic mice and age-matched C57BL/6 J mice were obtained from Beijing HFK Biotechnology (Beijing, China). The mice were 3, 6, and 12 months old. The mice were raised in cages with free access to breeding diet and water. The environment was maintained at 21–23°C, 50–60% humidity, and 12-h light/dark cycle. APP/PS1 are double transgenic mice expressing a chimeric mouse/human amyloid precursor protein (Mo/HuAPP695-swe) and mutant human presenilin 1 (PS1-dE9).

### Cell culture

The hippocampal neuronal cell line (HT22) was obtained from Procell (CL-0697, Procell, Wuhan, Hubei, China). HT22 was propagated in DMEM (10569010, Gibco, Shanghai, China) supplemented with 10% fetal bovine serum (S711-001S, Lonsera, Shanghai, China), 1% penicillin, and 1% streptomycin (S110JV, BasalMedia, Shanghai, China) at 37°C in a humidified 5% CO_2_ and 95% air atmosphere for 24 h. HT22 cells were treated with 20μM Aβ_25 − 35_ for 24 h, with or without followed by treating with 5 μM DAPT or 20μM EUK 134 for 24 h. Aβ_25 − 35_ (HY-P0128, MedChemExpress, Shanghai, China) was dissolved in double-distilled water, adjusted to 1 mM, and aggregated at 37°C for 5 days. DAPT (S2215, Selleck, Shanghai, China) and EUK 134 (S4261, Selleck, Shanghai, China) were dissolved in DMSO (D3871, Solarbio, Beijing, China) and adjusted to 1 mM.

### Quantitative real-time polymerase chain reaction

Two-milliliter blood samples were collected in an anticoagulation tube containing ethylenediaminetetraacetic acid. The mRNA of whole blood samples, mouse tissue, and HT22 cells was extracted using TRIzol (15596018, Invitrogen, Carlsbad, CA, USA). The OD260/280 was between 1.8 and 2.0. Reverse transcription was conducted using the Evo M-MLV RT Kit with gDNA Clean for qPCR (11705, Accurate Biology, Changsha, Hunan, China) and a Master cycler (Eppendorf, Hamburg, Germany). RT-PCR was performed using SYBR Green Premix Pro Taq HS qPCR Kit (11701, Accurate Biology, Changsha, Hunan, China) and real-time PCR system (QuantStudio5, Thermo Fisher Scientific, Marsiling, Singapore). Specific primers for TOM70, TOM40, TOM22, TOM20, and β-actin (Table 1) were generated using the BioSune Technology (Shanghai, China). The results were analyzed using the 2^−ΔΔCT^ method. Data were expressed as mRNA levels of TOM70, TOM70, TOM40, TOM22, and TOM20, normalized to β-actin mRNA levels in each sample.

**Table 1:** Oligonucleotide primer sets for RT-PCR.

**Name**	**Sequence (5'-3')**	**Length**	**Tm**
human TOM70F	GCATTGTACCGCCAGGCATA	20	57.45
human TOM70R	ATAGCCTTCGGCACACCTTG	20	57.45
mouse TOM70F	AGCTTCGAGCAAATGCCCTT	20	54.3
mouse TOM70R	GAGGCTGCCACTATTGTCCA	20	57.45
mouse TOM40F	TCACATACGTGGGGACGAAG	20	62
mouse TOM40R	GAGGCTGCCACTATTGTCCA	20	62
mouse TOM22F	ACGGAGATGTTTCCCGAGAG	20	62
mouse TOM22R	AAAGTATCTGCCGTTGCTGC	20	60
mouse TOM20F	CCCCAACTTCAAGAACAGGC	20	62
mouse TOM20R	CACACCCTTCTCGTAGTCACC	21	59.76
human β-actin F	CATGTACGTTGCTATCCAGGC	21	57.6
human β-actin R	CTCCTTAATGTCACGCACGAT	21	55.6
mouse β-actin F	GGCTGTATTCCCCTCCATCG	20	59.5
mouse β-actin R	CCAGTTGGTAACAATGCCATGT	22	55.8

### Western blotting

Hundred micrograms of protein per lane was subjected to 10% SDS–PAGE and transferred to polyvinylidene fluoride (PVDF) membranes (IPVH00010, Millipore, Darmstadt, Germany). The membranes were blocked with 5% skimmed milk (D8340, Solarbio, Beijing, China) and incubated with primary antibodies against TOM70 (1:1000 dilution, ab89624, Abcam, Shanghai, China; 1:1000 dilution, 14528-1-AP, Proteintech, Wuhan, Hubei, China), TOM40 (1:2000 dilution,18409-1-AP, Proteintech, Wuhan, Hubei, China), TOM22 (1:500 dilution, 11278-1-AP, Proteintech, Wuhan, Hubei, China), TOM20 (1:2000 dilution, 11802-1-AP, Proteintech, Wuhan, Hubei, China), β-actin (1:2000 dilution, 20536-1-AP, Proteintech, Wuhan, Hubei, China), or GAPDH (1:50000 dilution, 60004-1-AP, Proteintech Wuhan, Hubei, China) overnight at 4°C, followed by incubation with secondary species-specific HRP-coupled antibodies (1:5000 dilution, E-AB-1034, Elabscience Wuhan, Hubei, China) for 1 h at room temperature. Proteins were visualized using an ECL chemiluminescence kit (WBKIS0100, Millipore, Darmstadt, Germany) and a chemiluminescence imaging system (ProteinSimple, ChenQ, CA, USA). Bands were quantified using ImageJ software.

### Immunofluorescence

The mice were anesthetized with 2% pentobarbital sodium and perfused transcardially with 20 ml of ice-cold 0.1M PBS and 20 ml of ice-cold 4% paraformaldehyde. The brains were removed, fixed in 4% paraformaldehyde, and incubated in 30% sucrose solution overnight at 4°C. Sagittal sections were cut on a freezing microtome at a thickness of 20 μm (Leica Biosystems Inc., Nussloch, Germany). The sections were treated with 1% Triton X-100 (ST795, Beyotime, Shanghai, China), and blocked with 5% normal goat serum (SL038, Solarbio, Beijing, China) for 1 h.

The treated cells were fixed in 4% paraformaldehyde for 20 min, treated with 0.5% Triton X-100 (ST795,Beyotime, Shanghai, China), and blocked with 5% normal goat serum (SL038, Solarbio, Beijing, China) for 1 h.

After blocking with 5% normal goat serum, mouse brain sections and HT22 cells were incubated with primary antibody against TOM70 (1:200 dilution, sc-390545, Santa Cruz, Dallas, Texas, USA) overnight at 4°C and incubated with secondary antibodies (1:500 dilution, ab150115, Abcam, Shanghai, China) for 1 h at room temperature. Finally, the nuclei were stained with DAPI (F6057, Sigma, Shanghai, China), and images were observed using fluorescence microscopy and confocal microscopy.

### Statistical analysis

Categorical variables were presented as frequency (percentage, %), and continuous variables were presented as mean ± standard error of the mean. The Kolmogorov–Smirnov test was used to determine data normality. Bivariate correlation test, partial correlation test, unpaired two-way Student's *t-*test, and paired two-way Student's *t-*test were used for data analysis. Values of *P* < 0.05, 0.01, and 0.001 were considered statistically significant. All statistical analyses were performed using SPSS (version 25.0; SPSS, Inc., Chicago, IL, USA) and GraphPad Prism8, San Diego, CA, USA) for Windows.

## Results

### Demographic and clinical characteristics of the study subjects

To confirm the diagnosis, the CDR, MMSE, MoCA, and MTA were applied to all patients. Demographic and clinical characteristics of the study participants are listed in Table 2. The scale scores of patients with AD, DLB, and PSD were significantly lower than those of the sex- and age-matched control groups.

**Table 2:** Demographic and clinical characteristics of AD patients with age- and sex-matched controls.

	**Controls for AD (*N =* 40)**	**AD (*N =* 40)**	**Controls for DLB (*N =* 25)**	**DLB (*N =* 25)**	**Controls for PSD (*N =* 35)**	**PSD (*N =* 35)**
Age(years)	70.87 ± 7.33		78 ± 8.16		75 ± 6.67	
Sex: Male	55.33		60.00		71.43	
N (%)					
CDR	0.00 ± 0.00	2.03 ± 0.85***	0.00 ± 0.00	2.24 ± 0.77***	0.00 ± 0.00	2.08 ± 0.65***
MMSE	28.80 ± 1.25	15.00 ± 6.74***	28.12 ± 1.12	14.80 ± 6.51***	28.76 ± 1.24	13.65 ± 7.01***
MoCA	24.10 ± 1.21	12.00 ± 6.44***	24.34 ± 1.26	13.26 ± 6.93***	25.01 ± 1.28	11.74 ± 6.65***
MTA	0.30 ± 0.47	2.43 ± 1.13***	-	-	-	-

N, number; MMSE, Mini-Mental State Examination; MoCA, Montreal Cognitive Assessment; CDR, Clinical Dementia Rating; MTA, medial temporal lobe atrophy scale. ^***^P < 0.001.

### TOM70 mRNA level was reduced in peripheral blood of AD

**Figure 1:**
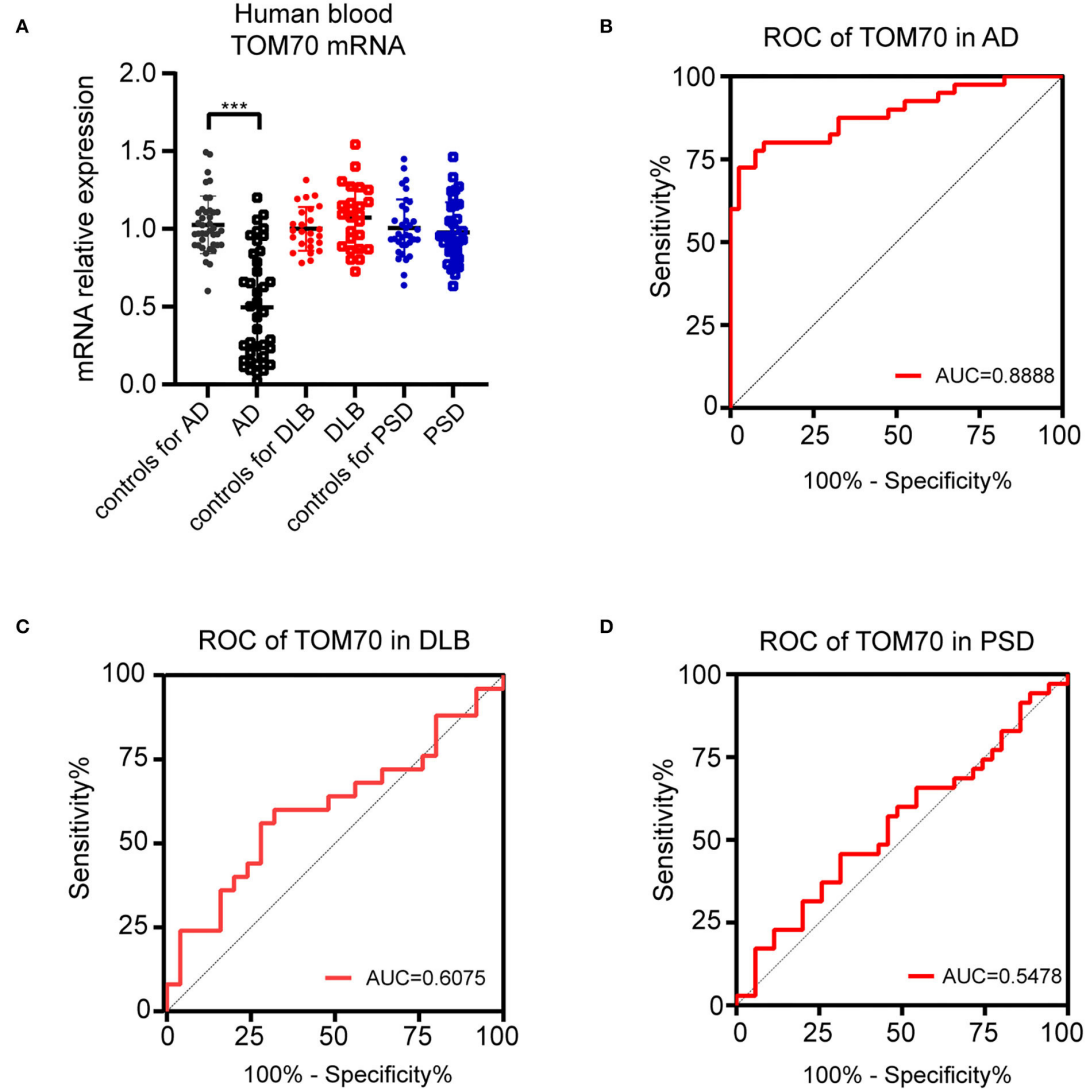
TOM70 levels in AD, DLB, and PSD. TOM70 mRNA levels from peripheral blood were significantly decreased in AD patients, as compared to normal groups. **(A)** TOM70 mRNA levels in peripheral blood of AD, DLB, and PSD patients with age- and sex-matched controls. **(B)** ROC curve of TOM70 in AD. **(C)** ROC curve of TOM70 in DLB. **(D)** ROC curve of TOM70 in PSD. Data are expressed as fold change compared with β-actin mRNA levels. Paired Student's *t-*test. ***P < 0.001.

To evaluate the TOM70 expression in AD patients, we tested the mRNA levels of TOM70 in peripheral blood. The data indicated that TOM70 mRNA levels were significantly decreased in AD patients, as compared to those in the normal group (Figure 1A, fold change = 2.20). However, we did not find significant differences between the DLB, PSD, and control groups.

To analyze the specificity and sensitivity of TOM70 in distinguishing dementia patients from controls, we performed ROC analysis. In AD, TOM70 levels in peripheral blood featured significantly high areas under the curve (AUCs), which far exceeded the random chance (AUC of 0.5) (Figure 1B, AUC = 0.8888). However, the AUCs of TOM70 levels in the DLB (Figure 1C, AUC = 0.6075) and PSD (Figure 1D, AUC = 0.5478) groups were not significant in the peripheral blood samples. This indicates that peripheral blood TOM70 has better specificity and sensitivity in patients with AD.

### Decrease in TOM70 levels correlates with the progression of AD

**Figure 2:**
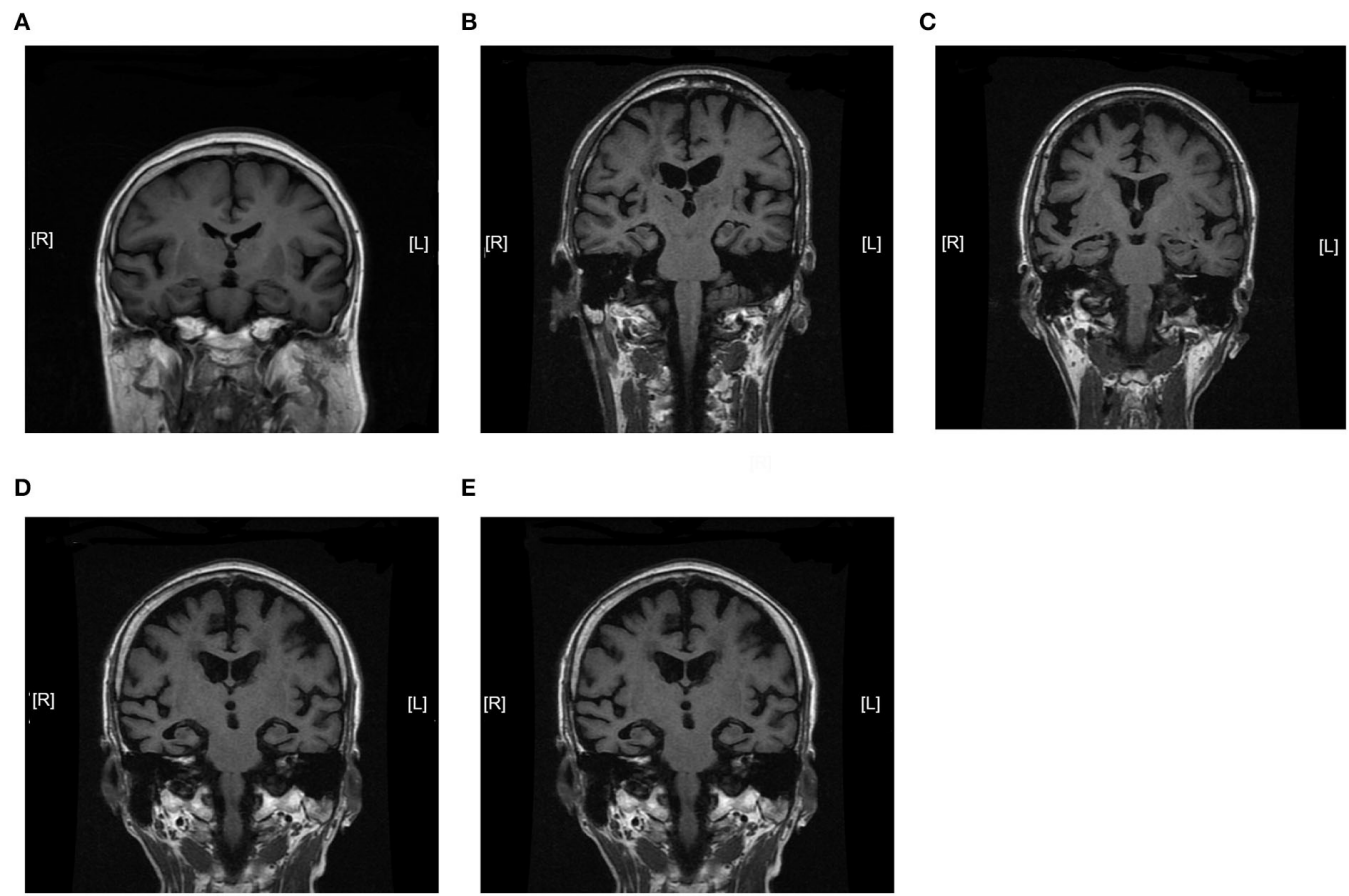
Magnetic resonance imaging coronal view of the hippocampus in T1-weighted images. MTA scores were used to assess AD progression. The degree of MTA correlates with AD severity. **(A)** MTA = 0. **(B)** MTA = 1. **(C)** MTA = 2. **(D)** MTA = 3. **(E)** MTA = 4. MTA: medial temporal lobe atrophy scale. MTA scores range from 0 to 4.

**Figure 3:**
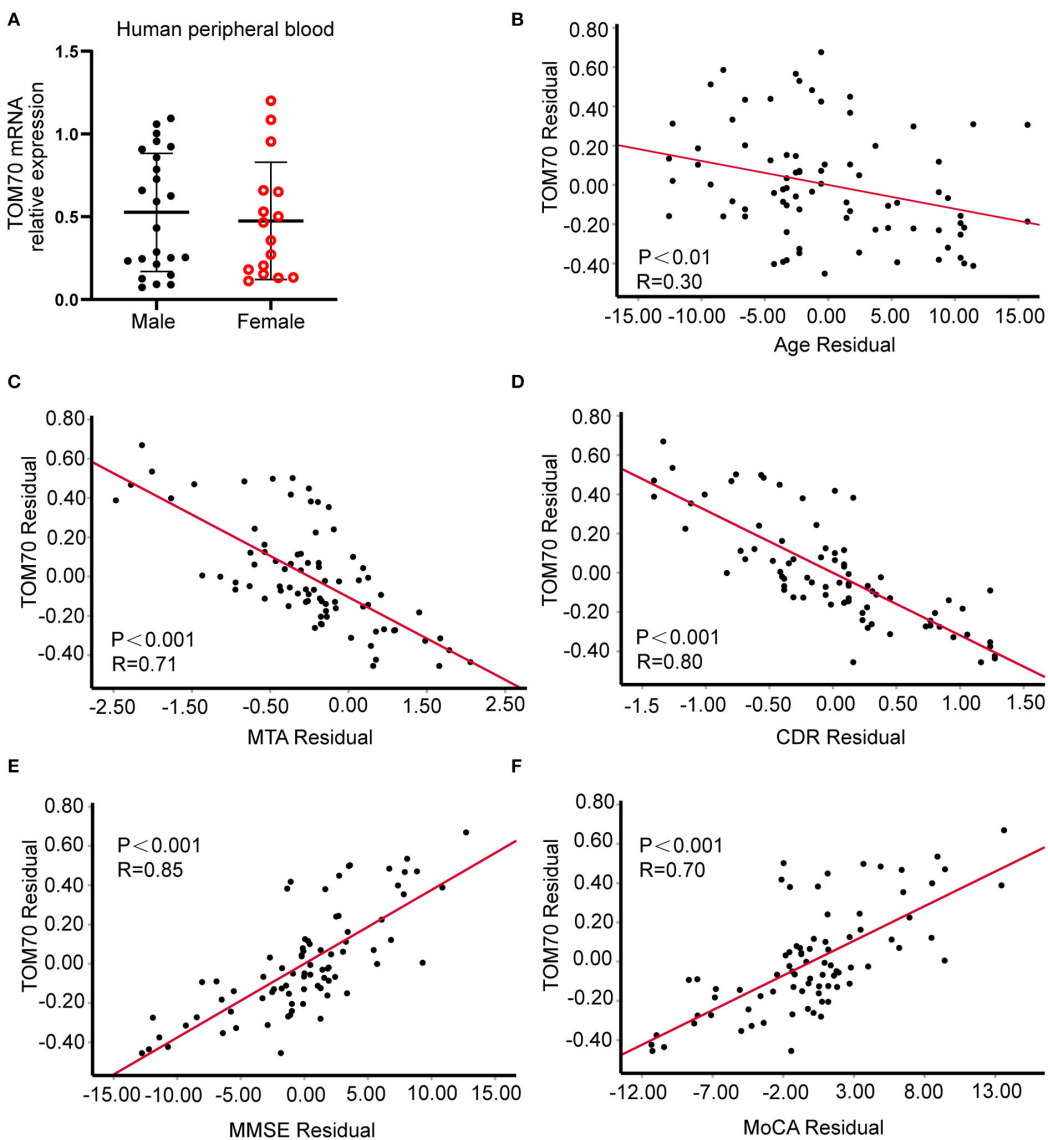
Partial correlation between TOM70 and variables. CDR score positively correlates with AD severity. MMSE and MoCA scores negatively correlate with AD severity. **(A)** A scatter plot of TOM70 per sex group. **(B)** Correlation of TOM70 and age adjusted for diagnosis and sex. **(C)** Partial correlation between Tom70 and MTA score adjusted for age, diagnosis, and sex. **(D)** Partial correlation between Tom70 and CDR score adjusted for age, diagnosis, and sex. **(E)** Partial correlation between Tom70 and MMSE score adjusted for age, diagnosis, and sex. **(F)** Partial correlation between Tom70 and MoCA score adjusted for age, diagnosis, and sex. *n* = 40 (AD) and 40 (controls).

We further analyzed the expression of TOM70 along with clinical characteristics in patients with AD. Medial temporal lobe atrophy scale (MTA) could be assessed quickly and easily with plain MRI films (Scheltens et al., 1992). In AD, the degree of MTA correlates with AD severity and can be used to predict dementia progression (Visser et al., 2002; Geroldi et al., 2006; Wattjes et al., 2009). In our study, we used MTA scores to assess AD progression. Figure 2 shows MTA scores of 0–4 of AD patients. We detected a correlation between TOM70 and sex, age, neuropsychological test scores and MTA scores. Partial correlation models are presented in Table 3. The scatter plots of the partial correlations are shown in Figure 3. There was no correlation between TOM70 level and sex (Figure 3A). There was a negative correlation between the TOM70 level and age (Figure 3B). The TOM70 mRNA level was negatively correlated with MTA score (Figure 3C) and CDR score (Figure 3D) and positively correlated with MMSE score (Figure 3E) and MoCA score (Figure 3F). MMSE and MoCA scores were negatively correlated with AD progression, whereas CDR and MTA scores were positively correlated with AD progression. Our data indicate that the decreased expression of TOM70 in peripheral blood correlated with the progression of AD.

**Table 3:** Partial correlation between TOM70 and variables.

	**Variables**	**Correlation**	**Significance (two tailed)**
Model 1	TOM70 vs. age	−0.306	0.007
Model 2	TOM70 vs. MTA	−0.709	< 0.001
	TOM70 vs. CDR	−0.795	< 0.001
	TOM70 vs. MMSE	0.753	< 0.001
	TOM70 vs. MoCA	0.696	< 0.001

MMSE, Mini-Mental State Examination; MoCA, Montreal Cognitive Assessment; CDR, Clinical Dementia Rating; MTA, medial temporal lobe atrophy scale; Model 1 was adjusted for gender and diagnosis. Model 2 was adjusted for age, gender, and diagnosis.

### TOM70 level decreased in hippocampus and peripheral blood of APP/PS1 mice

**Figure 4:**
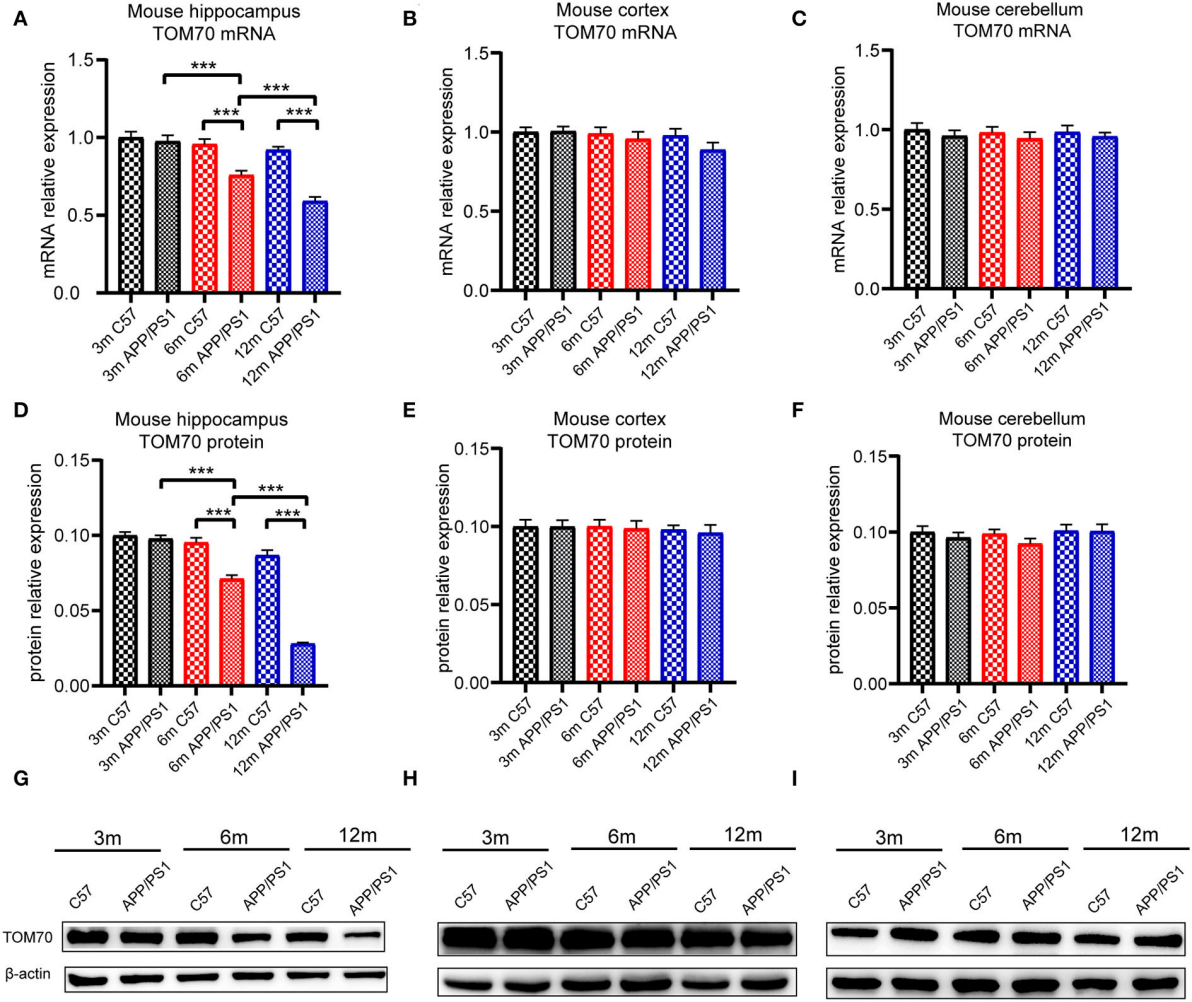
TOM70 level in mouse brain tissue. TOM70 mRNA and protein levels in mouse hippocampus, cortex, and cerebellum. TOM70 level was significantly reduced in the hippocampus of APP/PS1 mice at 6 and 12 months. **(A)** TOM70 mRNA level in the hippocampus of APP/PS1 and C57 mice aged 3, 6, and 12 months. **(B)** TOM70 mRNA level in the cortex of APP/PS1 and C57 mice aged 3, 6, and 12 months. **(C)** TOM70 mRNA level in the cerebellum of APP/PS1 and C57 mice aged 3, 6, and 12 months. **(D)** TOM70 protein levels in the hippocampus of APP/PS1 and C57 mice aged 3, 6, and 12 months. **(E)** TOM70 protein levels in the neocortex of APP/PS1 and C57 mice aged 3, 6, and 12 months. **(F)** TOM70 protein levels in the cerebellum of APP/PS1 and C57 mice aged 3, 6, and 12 months. **(G)** Representative western blotting bands of mouse hippocampus. **(H)** Representative western blotting bands of mouse cortex. **(I)** Representative western blotting bands of mouse cerebellum. Unpaired Student's *t-*test. *n* = 6 samples. m = month. Bar graph is expressed as mean ± SEM. *P < 0.05. **P < 0.01. ***P < 0.001.

**Figure 5:**
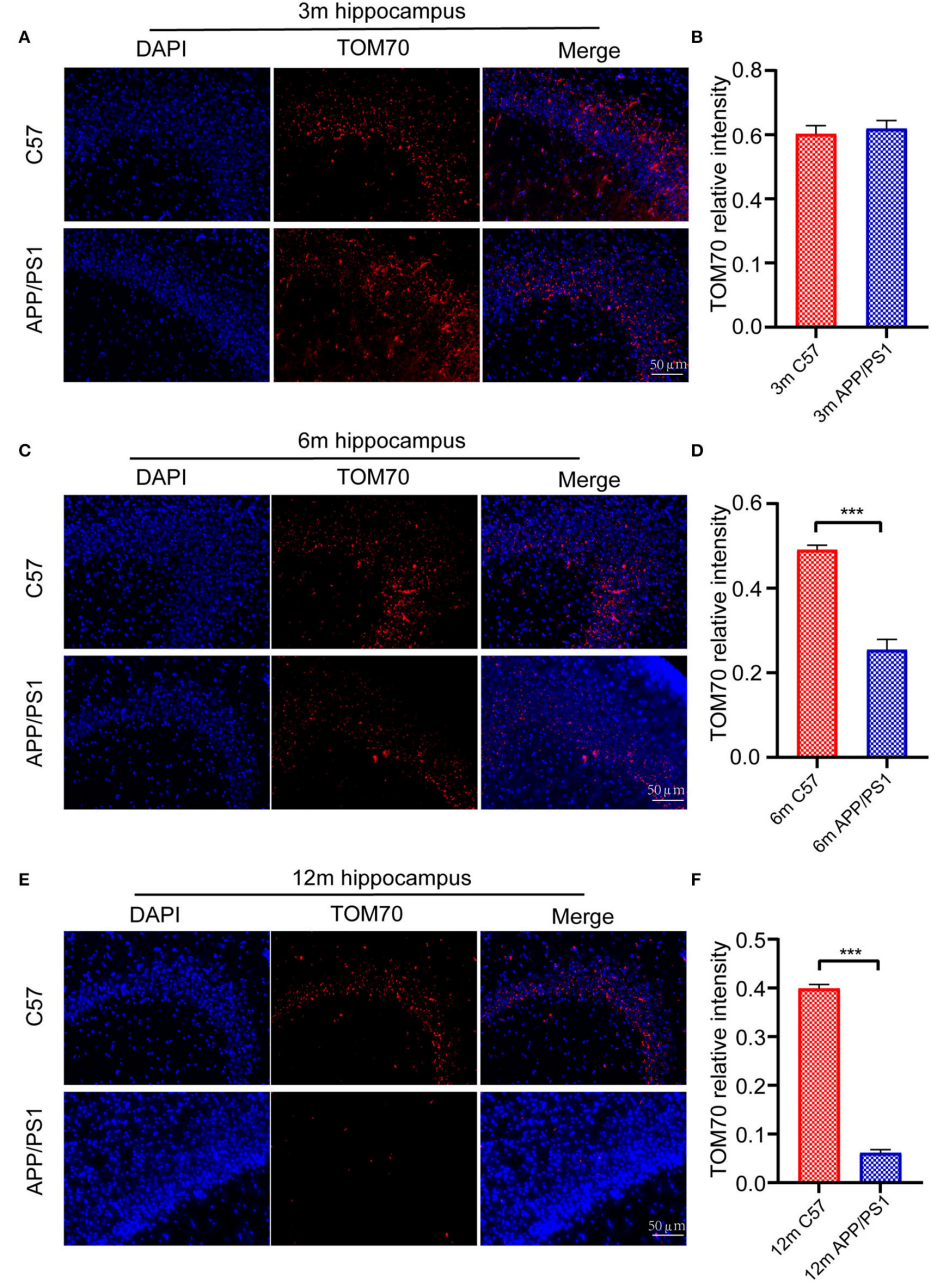
Immunofluorescence assay for mouse hippocampus. Fluorescence intensity of TOM70 was significantly decreased in the hippocampus of APP/PS1 mice at 6 and 12 months. **(A)** DAPI and TOM70 fluorescence for the hippocampus of 3-month C57 and APP/PS1 mice. **(B)** TOM70 fluorescence relative intensity in the hippocampus of APP/PS1 mice at 3 months. **(C)** DAPI and TOM70 fluorescence for the hippocampus of 6-month C57 and APP/PS1 mice. **(D)** TOM70 fluorescence relative intensity in the hippocampus of APP/PS1 mice at 6 months. **(E)** DAPI and TOM70 fluorescence for the hippocampus of 12-month C57 and APP/PS1 mice. **(F)** TOM70 fluorescence relative intensity in the hippocampus of APP/PS1 mice at 12 months. Scale = 50μm. The data are mean ±SEM (*n* = 3). ***P < 0.001.

**Figure 6:**
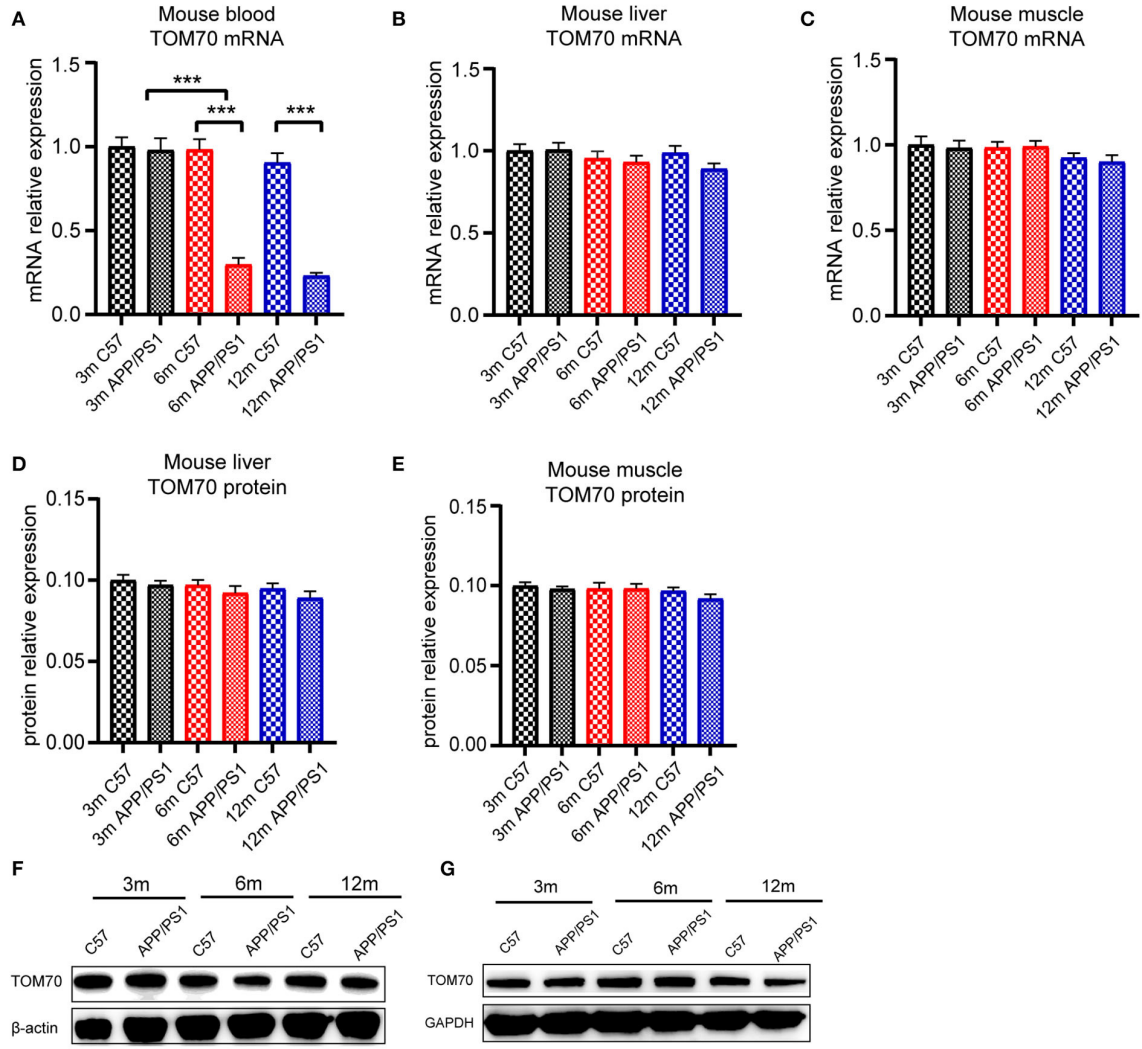
TOM70 level in mouse peripheral organs. TOM70 levels in mouse peripheral blood, liver, and muscle. TOM70 level was significantly reduced in the blood of APP/PS1 mice at 6 and 12 months. **(A)** TOM70 mRNA level in the peripheral blood of APP/PS1 and C57 mice aged 3, 6, and 12 months. **(B)** TOM70 mRNA level in the liver of APP/PS1 and C57 mice aged 3, 6, and 12 months. **(C)** TOM70 mRNA level in the skeletal muscle of APP/PS1 and C57 mice aged 3, 6, and 12 months. **(D)** TOM70 protein level in the liver of APP/PS1 and C57 mice aged 3, 6, and 12 months. **(E)** TOM70 protein level in the skeletal muscle of APP/PS1 and C57 mice aged 3, 6, and 12 months. **(F)** Representative western blotting bands of mouse liver. **(G)** Representative western blotting bands of mouse muscle. Unpaired Student's *t-*test. *n* = 6 samples. m = month. Bar graph is expressed as mean ± SEM. ***P < 0.001.

To test the TOM70 expression in APP/PS1 mice at different months, we examined TOM70 mRNA and protein expression levels in the peripheral blood, brain (including hippocampus, cortex, and cerebellum), liver, and skeletal muscle of 3-, 6-, and 12-month-old APP/PS1 mice, while we used C57 mice as a control. Our data showed that TOM70 mRNA levels were significantly reduced in the hippocampus of APP/PS1 mice at 6 and 12 months of age and correlated with age (Figure 4A). However, TOM70 mRNA levels in the cortex (Figure 4B) and cerebellum (Figure 4C) of the APP/PS1 mice did not decrease. TOM70 protein levels were significantly reduced in the hippocampus of APP/PS1 mice at 6 and 12 months of age and correlated with age (Figure 4D). In contrast, TOM70 levels in the cortex (Figure 4E) and cerebellum (Figure 4F) of the APP/PS1 mice did not decrease. Representative western blotting bands of mouse hippocampus, cortex and cerebellum are shown in Figures 4G–I. Immunofluorescence showed that TOM70 levels in the hippocampus of APP/PS1 mice were reduced at 6 and 12 months of age (Figure 5). As mitochondria are distributed in all organs, we further examined TOM70 expression levels in the peripheral blood, skeletal muscle, and liver of APP/PS1 mice. We demonstrated that TOM70 expression levels were significantly lower in the peripheral blood of the APP/PS1 mice (Figure 6A). TOM70 mRNA levels in the liver (Figure 6B) and muscle (Figure 6C) of the APP/PS1 mice did not decrease. TOM70 protein levels in the liver (Figure 6D) and muscle (Figure 6E) of the APP/PS1 mice did not decrease. Representative western blotting bands of mouse liver and muscle are shown in Figures 6F,G.

### TOM70 level decreased in Aβ_25 − 35_-treated HT22 cells

**Figure 7:**
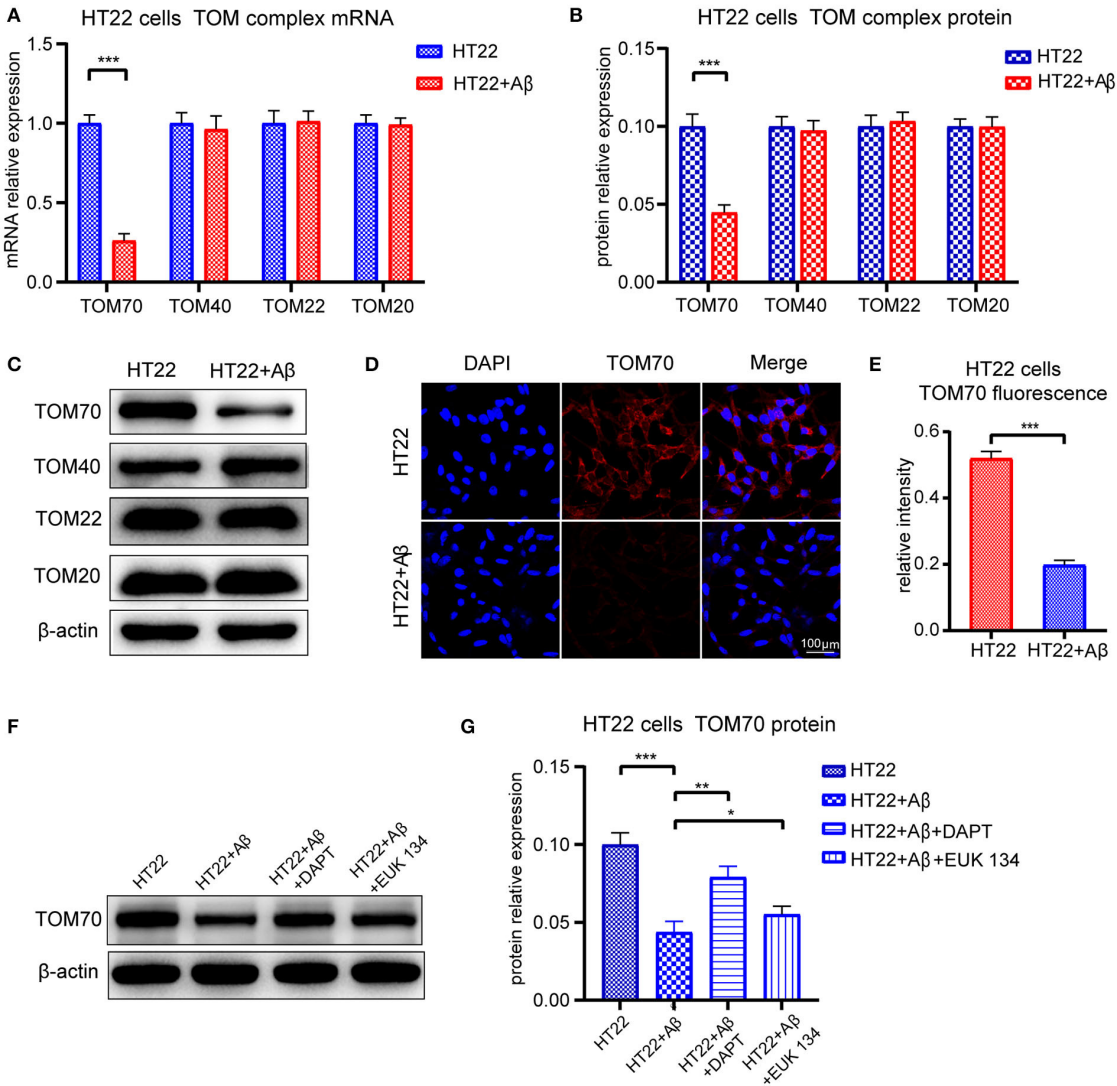
Expression of TOM complex in HT22 cells treated with Aβ for 24 h. Cells were treated with 20μM Aβ_25 − 35_ for 24 h, with or without followed by treating with 5μM DAPT or 20μM EUK 134 for 24 h. **(A)** TOM70, TOM40, TOM22, and TOM20 mRNA levels in HT22 cells after 24-h treatment with Aβ_25 − 35_. **(B)** TOM70, TOM40, TOM22, and TOM20 protein levels in HT22 cells after 24-h treatment with Aβ_25 − 35_. **(C)** Representative bands of western blot of TOM complex in Aβ2535-treated HT22 cells. **(D)** TOM70 fluorescence of HT22 cells treated with Aβ_25 − 35_ for 24 h. **(E)** TOM70 fluorescence relative intensity in HT22 cells after 24-h treatment with Aβ_25 − 35_. **(F)** Representative bands of western blot of TOM70 in Aβ_25 − 35_, DAPT, or EUK 134-treated HT22 cells. **(G)** TOM70 protein level in Aβ_25 − 35_, DAPT, or EUK 134-treated HT22 cells. *P < 0.05, **P < 0.01, ***P < 0.001.

To test whether TOM70 and other TOM components expression is affected by Aβ, we examined TOM70, TOM40, TOM22, and TOM20 mRNA and protein expression levels in Aβ_25 − 35_-treated HT22 cells. Our data showed that in HT22 cells, TOM70 mRNA (Figure 7A) and protein levels (Figure 7B) were significantly reduced after Aβ_25 − 35_ treatment. However, other TOM components expression was not reduced after Aβ_25 − 35_ treatment. Representative western blotting bands of TOM components are shown in Figure 7C. Immunofluorescence showed that TOM70 levels in Aβ_25 − 35_-treated HT22 cells were lower than those in untreated HT22 cells (Figures 7D,E). To validate that the reduced expression of TOM70 is associated with Aβ, we performed rescue experiments. DAPT is a γ-secretase inhibitor (Dovey et al., 2001), which has been used in many studies to inhibit Aβ production (Nasoohi et al., 2012; Yao et al., 2021). EUK 134 is a synthetic superoxide dismutase (SOD)/peroxidase analog with antioxidant activity and inhibitory effect on Aβ (Baker et al., 1998; Jekabsone et al., 2006). TOM70 protein levels were significantly reduced after Aβ_25 − 35_ treatment, while after being treated with DAPT or EUK 134 for another 24 h, the TOM70 expression was increased compared with Aβ-treated HT22 cells (Figures 7F,G).

## Discussion

Currently, methods for diagnosing AD and determining its progression are limited. Blood tests are less invasive, more convenient, and less expensive. Therefore, finding more blood biomarkers is urgently needed for Alzheimer's disease. Studies have shown that the amyloid precursor protein (APP) is blocked in TOM channels in the brain tissue of AD patients (Devi et al., 2006). TOM70, as a key protein, is involved in mitochondrial compromised protein import response (mitoCPR), which relieves the blockage of mitochondrial proteins on TOM channels (Weidberg and Amon, 2018). However, TOM70 level is reduced in the brain tissue of AD patients (Chai et al., 2018; Leal et al., 2020). Therefore, we speculated that TOM70 may be involved in the pathogenesis of AD. In this study, we found TOM70 levels reduced blood and hippocampus in APP/PS1 mice. Further, we collected peripheral blood from AD patients and found that the mRNA level of TOM70 was shown to be declining in the blood samples of AD patients, which is likewise connected with the progression of clinical stages. However, there was no significant difference in TOM70 levels in DLB and PSD patients, further demonstrating the relevance of TOM70 and AD.

*In vitro*, the TOM70 levels in HT22 cells decreased after amyloid beta (Aβ) treatment. One of the pathological features of AD is deposition of Aβ (Murphy, 2010). Aβ is widely expressed in the brain and peripheral organs, and peripheral Aβ may account for the large proportion of total Aβ (Roher et al., 2009). It has been observed in human and animal models that brain-derived Aβ peptides can be transported from the brain to peripheral blood (Roberts et al., 2014; Xiang et al., 2015). The role of peripheral Aβ in the progression of AD is unclear, but our study showed that only peripheral blood and hippocampal TOM70 were reduced in APP/PS1 mice, suggesting that the reduced TOM70 expression is involved in the pathogenesis and progression of AD. Studies have shown that Aβ produced by blood cells can affect AD progression (Sun et al., 2021). In addition, studies have shown that the liver can reduce Aβ production or peripheral Aβ levels (Bassendine et al., 2020). We hypothesized that as the hippocampus and peripheral blood deposit more Aβ and have lower Aβ scavenging ability, they are involved in the early stage of AD and hence show a decrease in TOM70. In addition, the level of TOM70 in peripheral blood, as well as in the brain, decreases with the progression of the disease. Therefore, we speculated that peripheral blood TOM70 levels may be a potential biomarker for the diagnosis and assessment of AD progression.

Another interesting finding of our study is that TOM70 levels are negatively correlated with age, which is one of the risk factors for AD (Armstrong, 2019). In addition, in the hippocampal tissues of mice, the expression of TOM70 decreased with increasing age. This may be related to mitochondria and aging. Few studies have focused on the relationship between changes in TOM function and aging, but some studies have found that the change in the TOM component expression may be the basis of oxidative phosphorylation dysfunction (Chai et al., 2018), which indicates that the change in TOM may be associated with aging by affecting mitochondrial function. In view of the correlation between age and TOM70 level, we excluded the influence of sex and age when we analyzed the correlation between TOM level and other variables, which also made the results more accurate.

In this study, we found that TOM70 levels were decreased in the hippocampus and peripheral blood of patients with AD. This finding suggests that decreased TOM70 levels may be related to the mechanism of AD and may be used as a potential biomarker for the diagnosis and progression of AD. However, the relatively small sample size of the enrolled subjects and the lack of further mechanistic studies at the molecular level are limitations of this study. We will continue our study by increasing the sample size in future cohort studies, and the molecular mechanisms underlying TOM70 changes in patients with AD will be emphasized in our further investigation. In conclusion, the results of our study showed that the expression levels of the TOM complex components changed in AD patients, which points us toward the direction for studying the mechanism of mitochondrial abnormalities in AD.

## Data availability statement

The original contributions presented in the study are included in the article/supplementary material, further inquiries can be directed to the corresponding author/s.

## Ethics statement

The studies involving human participants were reviewed and approved by the Institutional Review Board of the Second Hospital of Shandong University. The patients/participants provided their written informed consent to participate in this study. The animal study was reviewed and approved by the Institutional Review Board of the Second Hospital of Shandong University. Written informed consent was obtained from the individual(s) for the publication of any potentially identifiable images or data included in this article.

## Author contributions

LX designed research. XC analyzed data and wrote the paper. XC, YC, and XS performed research. ZX, ZZ, SX, PW, and JB collected blood samples. All authors contributed to the article and approved the submitted version.

## Funding

This work was supported by the National Natural Science Foundation of China (81901106 and 81870848), the Fundamental Research Funds of Chinese Academy of Medical Sciences (2019-RC-HL-026), Shandong University Multidisciplinary Research and Innovation Team of Young Scholars (2020QNQT019), Shandong Provincial Natural Science Foundation China (ZR2015HM024) (2019GSF108066), IIFDU, and SRF for ROCS, SEM.

## Conflict of interest

The authors declare that the research was conducted in the absence of any commercial or financial relationships that could be construed as a potential conflict of interest.

## Publisher's note

All claims expressed in this article are solely those of the authors and do not necessarily represent those of their affiliated organizations, or those of the publisher, the editors and the reviewers. Any product that may be evaluated in this article, or claim that may be made by its manufacturer, is not guaranteed or endorsed by the publisher.
